# A flow cytometric method for measuring and isolating mammary epithelial cells from bovine milk

**DOI:** 10.3168/jdsc.2021-0135

**Published:** 2021-08-26

**Authors:** A.J. Lengi, M. Makris, B.A. Corl

**Affiliations:** 1 Department of Dairy Science, Virginia Tech, Blacksburg 24061-0315; 2 Flow Cytometry Laboratory, Virginia-Maryland College of Veterinary Medicine, Virginia Tech, Blacksburg 24061

## Abstract

Sampling frequent time points of mammary signaling pathways is not possible with tissue biopsies. We have validated a flow cytometry and cell sorting procedure for isolating live bovine mammary epithelial cells from somatic cell populations in milk using butyrophilin 1A1 as a marker for mammary epithelial cells and CD45 as a marker for hematopoietic cells. Hoechst 33342 staining and propidium iodide exclusion were used to select for nucleated live cells. Positive selection of butyrophilin (BTN)-expressing cells was performed by fluorescence-activated cell sorting. Quantitative real-time PCR performed on mRNA isolated from these cells showed a 226-fold increase in κ-casein (*CSN3*) mRNA expression in BTN single-positive cells compared with unsorted cells, whereas CD45 single-positive cells showed a significant decrease. A negative selection strategy for cells not expressing the hematopoietic cell marker CD45 also resulted in a cell population with a 196-fold increase in *CSN3* mRNA expression compared with unsorted cells. We found no enrichment of *CSN3* mRNA expression after sorting cells using cytokeratin antibodies. The noninvasive assays described here allow for daily or more frequent sampling time points for measurement of mammary epithelial cells during the course of lactation.

## Highlights

• Flow cytometry using an antibody against butyrophilin allows quantification of mammary epithelial cells in milk.
• Sorting butyrophilin-positive or CD45-negative cells isolates epithelial cells from milk.
• Selection for cytokeratin was not effective in this flow cytometry application.

## Graphical Abstract

**Figure:**
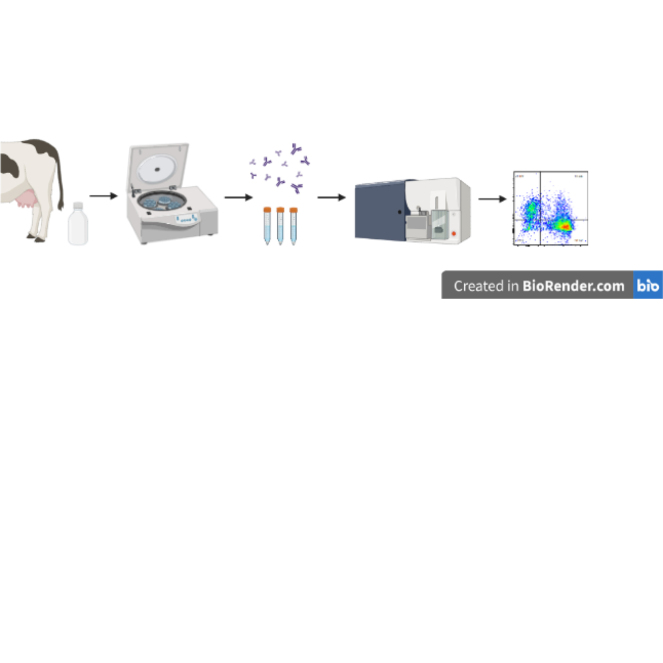
**Summary:** This technical note describes a method for isolating mammary epithelial cells from cow milk. Milk somatic cells were concentrated by centrifugation and the mammary epithelial cells were labeled with a butyrophilin antibody. Quantification and isolation were accomplished by flow cytometry and fluorescence activated cell sorting.

Traditionally, assessing the activation state and timing of signaling pathways in the mammary gland during lactation has required collecting mammary tissue biopsies. Because of the invasive nature of this procedure, only a small number of time points can be examined over the course of lactation, potentially missing changes in expression of genes that occur acutely or transiently. One possible less invasive method would be to use the mammary epithelial cells (**MEC**) that are exfoliated or extruded into milk, allowing much more frequent sampling. In cows, however, epithelial cells make up only a portion of the total somatic cells found in milk, with the remainder being mostly cells of hematopoietic origin: macrophage, monocytes, T cells, and granulocytes such as neutrophils (Li et al., 2015; Alhussien and Dang, 2018a). The numbers and relative proportions of these cells can vary tremendously from cow to cow, and even within a cow over the course of lactation or as health status changes (Alhussien and Dang, 2018a,b). Therefore, to be able to compare between samples, it is first necessary to enrich or purify MEC from the somatic cell population found in milk.

Previously, some researchers used cytokeratin antibodies in magnetic bead separation protocols to isolate MEC from the milk somatic cell population (Boutinaud et al., 2008, 2015). Cytokeratins, also commonly known as keratins, are a large family of type I and type II intermediate filament proteins primarily expressed in epithelial cells (Jacob et al., 2018). Intermediate filaments are components of the cytoskeleton that function to maintain the internal 3-dimensional cell structure (Herrmann et al., 2007). Different subsets of keratins are expressed in different epithelial cell populations (Karantza, 2011).

Several antibodies have been applied to the isolation or quantification of bovine milk MEC. Early work with magnetic bead separation techniques involved the use of an antibody against cytokeratin 8 (Clone K8.13), and researchers found significant correlations between levels of mammary transcripts isolated from their enriched MEC population and mammary balance data for glucose in feed-restricted cows (Boutinaud et al., 2008). The antibody clone used in that work, however, is no longer available. Another group used an antibody against cytokeratin 18 (Clone KS-B17.2) in a similar magnetic bead separation protocol (Krappmann et al., 2012). That study found no significant correlation between specific gene expression in the isolated cell population and udder tissue, leading the authors to speculate that the cells isolated from milk may have reduced viability or integrity. The inability to select for live cells is a limitation of magnetic bead separation protocols. More recent studies with magnetic beads used an antibody that recognizes high-molecular-weight cytokeratins 1, 5, 10, and 14 (**HMW CK**, Clone34βE12) to measure MEC exfoliation rate (Herve et al., 2017).

In this paper, we describe a flow cytometry method using fluorescence-activated cell sorting (**FACS**) to obtain an enriched population of live bovine MEC that can be used to study mRNA expression of critical genes involved in milk synthesis pathways, as well as studying the process of mammary cell loss into milk during lactation. The vast majority of somatic cells found in milk are known to be either epithelial cells, which should express cytokeratin, or CD45-expressing hematopoietic cells. CD45, also known as common leukocyte antigen or Ly-5, is a transmembrane protein tyrosine phosphatase that is expressed on the surface of all nucleated cells of hematopoietic origin, and it has long been used as a marker for hematopoietic cells (Donovan and Koretzky, 1993; Woodford-Thomas and Thomas, 1993).

We tested antibodies previously used in magnetic bead separation assays in a FACS protocol. All procedures involving cows were completed with Virginia Tech Institutional Animal Care and Use Committee approval, and milk was sampled from 3 multiparous mid- to late-lactation cows for each experiment. To isolate somatic cells from milk, approximately 3.8 L of milk from each cow, containing a final concentration of 0.5 m*M* EDTA, was centrifuged at 600 × *g* for 10 min. The resulting pellet was washed once with Dulbecco's PBS (**DPBS**) containing a final concentration of 0.5 m*M* EDTA and resuspended in red blood cell lysis buffer (154 m*M* NH_4_Cl, 10 m*M* KHCO_3_, 0.1 m*M* EDTA) for 15 min at room temperature. To remove cell clumps and noncellular debris, cells were filtered sequentially through 100- and 40-µm cell strainers (Genesee Scientific). Cells were then counted using a hemocytometer; 2 × 10^6^ cells were added to each tube for staining for flow cytometry or 2 × 10^7^ cells for FACS.

Optimal antibody concentrations were first determined by performing a series of single-color titrations. Primary antibodies and concentrations used in this study were as follows: CD45 clone CACTB51A, Kingfisher Biotech, 3.1 ng/µL; HMW CK clone34βE12, Novus Biologicals, 2.5 ng/µL; cytokeratin 18 clone KS-B17.2, Sigma Aldrich, 5 ng/µL. Secondary antibodies and concentrations used in this study are as follows: rat anti-mouse IgG_2a_-phycoerythrin (**PE**) clone SB84a, Southern Biotech Associates, 1.0 ng/µL; goat anti-mouse IgG_1_-AlexaFluor 488 (**AF488**), polyclonal, Southern Biotech Associates, 1.25 ng/µL. Cells were incubated with primary antibodies at the indicated concentrations for 40 to 60 min in 100 µL of Cell Staining Buffer (BD Biosciences) at room temperature and protected from light. Cells were washed with DPBS, collected by centrifugation at 600 × *g* for 10 min, and then incubated in appropriate secondary antibody at the indicated dilutions for 40 to 60 min in 100 µL of Cell Staining Buffer at room temperature and protected from light. Cells were washed as before, and then resuspended in Hoechst 33342 (Invitrogen, 10 µg/mL) and propidium iodide (**PI**; BD Biosciences, 5 µg/mL) for 40 to 60 min in 100 µL of DBPS at room temperature and protected from light. After a final wash, cells were resuspended in 100 µL of Cell Staining Buffer and analyzed by flow cytometry or used for FACS on a BD FACSAria Fusion Flow Cytometer.

For the first experiment, cells were double labeled with antibodies against CD45 (PE), and HMW CK conjugated to AF488. Using the BD FACSAria Fusion Cell Sorter, an initial gate was drawn based on FSC-A and SSC-A, after which doublet exclusion was performed to eliminate aggregates using FSC-H/W and SSC-H/W parameters. Live, nucleated cells were selected by gating sequentially on Hoechst-positive and PI-negative cells, after which cells were sorted into HMW CK+CD45− and HMW CK−CD45+ populations.

RNA was isolated from these 2 populations and from an unsorted aliquot of milk somatic cells, using RNAzol RT (Molecular Research Center Inc.) according to the manufacturer's instructions, and quantitated using a NanoDrop spectrophotometer. Equal amounts of RNA were reverse transcribed into cDNA using the Omniscript RT kit (Qiagen) according to the manufacturer's instructions. The BestKeeper algorithm (Pfaffl et al., 2004) was used to choose the top 2 most appropriate endogenous control genes, *RPLP0* and *PPIA*, from a panel of 11 candidates using cDNA from lactating bovine mammary tissue and bovine spleen. Quantitative real-time PCR was performed using the QuantiTect SYBR Green PCR Kit (Qiagen) according to the manufacturer's instructions. κ-Casein (*CSN3*) was used as a hallmark transcript for MEC. All primer pairs were designed to span at least one intron and generated a single product on melt curve analysis. Primer sequences, product sizes (Prod), and efficiencies (Eff) were as follows: *CSN3* forward (F): AGCCCACCTGAGATCAACAC, reverse (R): GCCGGATCTGTGAAAATCAT, Prod: 173 bp, Eff: 106.4; *RPLP0* F: TTACACCTTCCCACTTGCTG, R: TCCGACTCCTCCTTTGCTT, Prod: 150 bp, Eff: 102.4; *PPIA*: F: TCTGAGCACTGGAGAGAAAGG, R: ACCACCCTGGCACATAAATC, Prod: 82bp, Eff: 100.9. To validate enrichment, we used RNA isolated from unsorted cells from the same sample as the comparator. The PCR data were analyzed using the common base method (Ganger et al., 2017, 2020), and statistical analysis was completed using the Mixed procedure of SAS (v. 9.4; SAS Institute Inc.). The model included the fixed effect of treatment (cell type isolated by sorting) and the random effect of cow. When a significant treatment effect was detected, means were separated using Tukey's multiple comparison adjustment in the LSMeans statement.

While we found that CD45 single-positive cells had significantly reduced *CSN3* mRNA expression compared with unsorted cells, there was no significant enrichment for *CSN3* mRNA expression in the HMW CK single-positive cells compared with unsorted cells, indicating that this population of cells was not enriched for MEC (Figure 1A). We also attempted to perform FACS using an antibody against CK18 previously used in the literature (Krappmann et al., 2012). In this case, we were unable to obtain enough live single-positive cells for either CK18 or CD45 for RNA isolation, because nearly all cells positive for one marker were also positive for the other (Figure 1B and Table 1). Neither of these antibodies appears to be suitable for the isolation of MEC from milk somatic cells by FACS based on labeling and enrichment.

**Figure 1:**
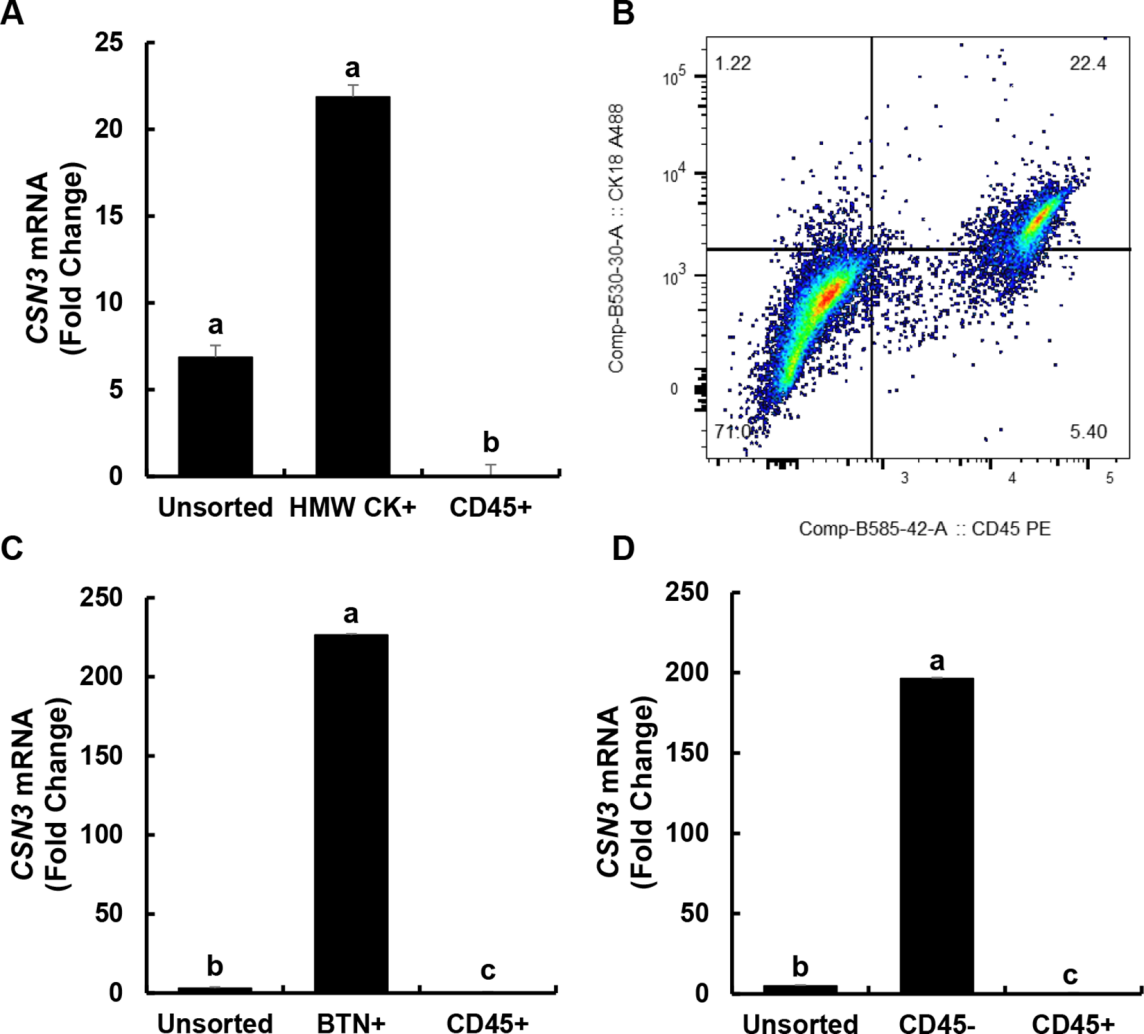
κ-Casein (*CSN3*) mRNA expression in milk somatic cells from lactating dairy cows sorted by fluorescence-activated cell sorting. (A) Unsorted cells compared with cells sorted using an antibody against bovine cytokeratin (HMW CK+) or bovine CD45 (CD45+). (B) Representative flow cytometry scatterplot showing staining for CD45-PE (phycoerythrin) on the x-axis and CK-18-AlexaFluor488 (A488) on the y-axis. (C) Unsorted cells compared with cells sorted using an antibody against bovine butyrophilin 1A1 (BTN+) or bovine CD45 (CD45+). (D) Unsorted cells compared with cells not expressing CD45 (CD45−) or expressing CD45 (CD45+). Values are least squares means and error bar is SEM (n = 3). Least squares means not sharing a common letter (a–c) are significantly different (*P* < 0.05).

**Table 1:** Cell population characteristics (%) of milk somatic cells labeled live or after being fixed and permeabilized

Population	Live	Fixed	SEM	*P*-value
CK18 antibody^1^				
BTN+ CK18− CD45−	1.23	0.17	0.46	0.24
CK18+ BTN− CD45−	4.52	7.35	1.35	0.21
CD45+ BTN− CK18−	0.11	0.42	0.09	0.14
BTN+ CD45+ CK18−	0.01	0.01	<0.01	0.18
CK18+ BTN+ CD45−	21.68	18.70	7.81	0.11
CK18+ CD45+ BTN−	60.09	58.64	8.40	0.51
CK18+ BTN+ CD45+	7.74	12.55	2.16	0.12
CK18− BTN− CD45−	4.16	1.30	0.89	0.01
HMW CK antibody^2^				
BTN+ HMW CK− CD45−	1.48	0.33	0.47	0.21
HMW CK+ BTN− CD45−	4.52	6.90	1.36	0.21
CD45+ BTN− HMW CK−	0.11	0.38	0.08	0.13
BTN+ CD45+ HMW CK−	0.82	1.43	0.34	0.31
HMW CK+ BTN+ CD45−	21.92	19.72	8.39	0.24
HMW CK+ CD45+ BTN−	60.05	55.73	9.50	0.20
HMW CK+ BTN+ CD45+	6.59	13.41	2.06	0.01
HMW CK− BTN− CD45−	4.13	1.19	0.85	0.01

1 Cells were labeled with antibodies against cytokeratin 18 (CK18), butyrophilin 1a1 (BTN), and CD45 and were positively labeled (+) or unlabeled (−).

2 Cells were labeled with antibodies against high-molecular-weight cytokeratins 1, 5, 10, and 14 (HMW CK), butyrophilin 1a1 (BTN), and CD45 and were positively labeled (+) or unlabeled (−).

Cytokeratins have a cytoplasmic expression profile and may not be accessible to antibodies unless cells are fixed and permeabilized before staining. Because we used PI to only include cells with intact membranes and exclude dead and dying cells from our sorted populations, we may have excluded the cells in which the cytokeratin antibodies would have had access to their targets. We therefore considered alternative MEC markers with cell surface expression. Butyrophilin 1A1 (**BTN**) is an integral acidic glycoprotein and a member of the immunoglobulin superfamily, structurally comprising exoplasmic immunoglobulin folds, a transmembrane anchor, and a cytoplasmic tail. The *BTN* gene is highly expressed in the secretory epithelium of the lactating mammary gland and has been shown to be essential for the regulated secretion of milk lipid droplets (Ogg et al., 2004). Previous work has shown that protein expression of BTN in the mammary gland is restricted to the apical surface of milk-secreting epithelial cells and the milk fat globule membrane, and not to other mammary cell types or epithelial tissues (Franke et al., 1981). These characteristics make BTN a good potential marker for MEC in the milk somatic cell population. We chose an antibody (BTN1A1 clone 2151C, Novus Biologicals, 7.0 ng/µL) that recognizes the extracellular domain of BTN conjugated to allophycocyanin (APC) and performed FACS analysis and real-time PCR as described above.

The results show that *CSN3* expression was significantly reduced in mRNA from CD45 single-positive cells compared with unsorted cells, as seen previously; however, in contrast to the results obtained with the HMW CK single-positive cells, BTN single-positive cells showed a dramatic increase in *CSN3* mRNA expression compared with unsorted cells (*P* < 0.05, Figure 1C). This suggests that BTN is a suitable marker for isolation of an enriched population of MEC from milk somatic cells.

We hypothesized that fixing and permeabilizing cells might allow for more specific selection of MEC using cytokeratin antibodies, although this would make these cells unsuitable for downstream applications requiring live cells. To determine whether fixing and permeabilizing cells would allow for sorting of MEC with cytokeratin antibodies, milk somatic cells were triple stained with BTN-APC, CD45-PE, and either HMW CK-AF488, or CK18-AF488 antibodies. A parallel set of cells were fixed and permeabilized before staining. Data were statistically analyzed using the Mixed procedure of SAS (v. 9.4; SAS Institute Inc.). The model included the fixed effect of treatment (live or fixed) and the random effect of cow. Table 1 shows a comparison of the staining for these markers in live versus fixed and permeabilized cells. Notably, for both of the cytokeratin antibodies, a large majority of the cells, around 60%, were double positive for cytokeratin and CD45, with no significant difference between live and fixed cell populations. Although there was also a substantial population double positive for cytokeratin and BTN (around 20% for both antibodies, with no difference between live and fixed), it does not appear that either cytokeratin antibody is specific for MEC because of the large amount of cross-reactivity with CD45+ cells, regardless of fixation state. Fixing and permeabilizing the cells had few significant effects on the percentages of stained cell populations: for the cells triple stained with antibodies against BTN, CD45, and CK18, there was a decrease in the percentage of triple-negative fixed cells versus live cells (*P* = 0.01). For the cells triple stained for BTN, CD45, and HMW CK, there was a similar decrease in the percentage of triple-negative fixed cells versus live cells, with a concomitant increase in the percentage of triple-positive fixed cells (*P* = 0.01).

Considering that the vast majority of somatic cells in milk are either CD45-expressing cells or MEC, which should not express CD45, we next examined whether a negative selection FACS strategy could be effective for generating an enriched population of MEC. For this experiment, cells were isolated as described above and sorted into CD45-positive versus CD45-negative populations. RNA isolation and real-time PCR were performed as before. Similar to the results obtained when sorting for BTN+ and CD45+ cells, *CSN3* expression was significantly reduced in mRNA from CD45-positive cells compared with unsorted cells, whereas CD45-negative cells showed a significant increase in *CSN3* mRNA expression compared with unsorted cells (*P* < 0.05, Figure 1D). This demonstrates that a negative selection strategy of removing CD45-positive cells is also effective for obtaining an enriched population of MEC.

This paper describes a new method for isolating MEC from bovine milk. In addition to achieving a greatly enriched population of MEC, the method offers greater selection capability than methods such as magnetic bead isolation, in that gates can be used to select further subsets of cells, such as using a viability stain to specifically select live cells that can be used for downstream applications to study gene expression pathways. This method allows for the comparison of live versus dead cells or of cells in different stages of apoptosis within a cell type.

Using cell sorting, we found that positive selection for BTN resulted in a 226-fold enrichment in *CSN3* mRNA compared with unsorted cells, while negative selection for CD45 resulted in a 196-fold enrichment. The current study did not directly compare this method of cell isolation from milk with methods used in other studies; however, using a cytokeratin 18 antibody-mediated magnetic bead isolation, one study found an approximately 4-fold increase in cytokeratin 18 (*KRT18*) mRNA expression compared with udder tissue (Krappmann et al., 2012), and another study using a cytokeratin 8 antibody-mediated magnetic bead isolation similarly found an approximately 4-fold increase in cytokeratin 8 (*KRT8*) mRNA expression in isolated cells compared with total milk somatic cells (Boutinaud et al., 2008).

The cytokeratin antibodies typically used in immunomagnetic bead protocols do not appear to be appropriate for this flow cytometry application. These antibodies are not validated by the manufacturer for use beyond immunohistochemistry and western blotting. In our flow cytometry application, they appeared to be highly cross reactive with hematopoietic cells, regardless of whether the cells were fixed and permeabilized. Cytokeratins are intracellular targets that require permeabilization to be accessible to antibodies, and therefore would be unsuitable for isolating live cells for downstream gene expression studies. Our experiments showed that sorting of milk somatic cells based on cytokeratin staining did not result in significant enrichment of MEC-specific expression of *CSN3* compared with an unsorted population of milk somatic cells. Using an antibody that binds to an extracellular target such as BTN allows for sorting of live cells because no fixing and permeabilizing is necessary. Further evidence supporting the use of BTN as a marker to isolate epithelial cells comes from a recent study using single cell sequencing with human milk somatic cells, which found that BTN is highly expressed in the subset of mature, secretory MEC, whereas cytokeratins 8 and 18 were more highly expressed in progenitor and maturing populations (Martin Carli et al., 2020). We confirmed that sorting of milk somatic cells based on BTN labeling resulted in a significant enrichment in mRNA expression of *CSN3* compared with unsorted cells.

The main limiting factors for determining how many samples could reasonably be processed in a day are the laboratory's capacity for centrifuging large volumes of milk and the time needed to sort the cells. For most of the samples we have processed, sufficient cell numbers were obtained by sorting for 1 h. One study found that the viability of milk somatic cells stored in PBS decreases from 39.5 to 35.7% after 24 h, whereas cells stored in milk do not exhibit a significant decline in viability even after 72 h (Li et al., 2015). Therefore, it is unlikely that adding a few extra hours to the length of the protocol to process additional samples would have a significant negative effect on the ability to select viable cells by FACS.

Although this method has not yet been tested using milk from mastitic cows, it is reasonable to expect that the increased influx of neutrophils and other immune cells into a mastitic gland would greatly dilute the percentage of MEC. This pitfall could be overcome by starting with a larger volume of milk and sorting for longer to obtain sufficient numbers of epithelial cells. Some studies, however, have found that disruption of the integrity of the mammary epithelium due to infection increases the percentage of MEC in milk (Wagner et al., 2009). More work needs to be done to compare the efficiency of this method in mastitic milk versus milk from healthy cows.

The vast majority of somatic cells in milk are known to be either MEC or hematopoietic cells. We show here that in addition to using positive selection to isolate MEC, a negative selection strategy of sorting CD45− cells was also highly effective and resulted in significant enrichment of *CSN3* mRNA expression compared with unsorted cells. These positive and negative FACS selection strategies can open new doors for frequent, repeated sampling of MEC that would not be possible with biopsies, and without any additional risk or stress to the animal. This noninvasive method can help improve our understanding of lactation biology by providing a homogeneous population of cells for study versus the heterogeneous and variable nature of tissue samples, and can allow for in vitro studies of cell function or for following responses to treatments at frequent time points to study acute changes in signaling pathways.
